# Synthesis and supramolecular self-assembly of glutamic acid-based squaramides

**DOI:** 10.3762/bjoc.14.180

**Published:** 2018-08-06

**Authors:** Juan V Alegre-Requena, Marleen Häring, Isaac G Sonsona, Alex Abramov, Eugenia Marqués-López, Raquel P Herrera, David Díaz Díaz

**Affiliations:** 1 Laboratorio de Organocatálisis Asimétrica. Departamento de Química Orgánica Instituto de Síntesis Química y Catálisis Homogénea (ISQCH) CSIC-Universidad de Zaragoza C/ Pedro Cerbuna 12, 50009 Zaragoza, Spain; 2 Institut für Organische Chemie, Universität Regensburg, Universitätsstr. 31, 93053 Regensburg, Germany; 3 Institute of Advanced Chemistry of Catalonia – Spanish National Research Council (IQAC–CSIC), Jordi Girona 18–26, 08034 Barcelona, Spain

**Keywords:** glutamic acid derivative, organogel, self-assembly, squaramide, supramolecular gel

## Abstract

We describe the preparation and characterization of two new unsymmetrical squaramide-based organogelators. The synthesis of the compounds was carried out by subsequent amine condensations starting from dimethyl squarate. The design of the gelators involved a squaramide core connected on one side to a long aliphatic chain and on the other side to a glutamic acid residue. The gelator bearing the free carboxylic groups showed a lower gelation capacity than its precursor diester derivative. Some selected gels were further studied by infrared spectroscopy, rheology and electron microscopy. Critical gelation concentrations and gel-to-sol transition temperatures were also determined for each case. In addition, the superior squaramide diester gelator was compared with an analogue triazole-based gelator in terms of critical gelation concentration, gelation kinetics and thermal phase transition.

## Introduction

Since their discovery, squaramides have gained importance across different fields from chemistry to biomedicine due to their synthetic versatility and wide applicability [1]. These compounds, formed by two amine units conjugated to an aromatic cyclobutenedione ring, can be easily synthesized from different derivatives of squaric acid and amines [1–4]. The possibility to fabricate chiral squaramide derivatives and their efficient hydrogen bond donor/acceptor ability has driven the pivotal role of these compounds in asymmetric catalysis and molecular recognition [5–6]. Besides, squaramides present a dual ability to recognize anions and cations through hydrogen bonding interactions, acting as ion sensors and transmembrane anion transporters [7]. This property has been crucial for the development of new drugs [8–9]. Moreover, these compounds have been recognized as bioisosters of ureas [10] exhibiting promising pharmacological properties [11] and being clinical candidates for the treatment of different diseases [1]. In addition, these compounds have shown relevance in other areas, including organic synthesis [12] and crystal engineering [13–17].

Despite their isosteric relationship with ureas, which have become key synthons in supramolecular chemistry [18–19], there are only a few reports on the formation of self-assembled supramolecular gels using squaramide derivatives [20–23]. Along this line, supramolecular or physical gels have received great attention during the last decade [24–25] due to their unique architectures and potential applications in many areas such as biomedicine (mainly hydrogels), health care and catalysis, among others [26–29]. In contrast to chemical gels [30], physical gels are typically made of low-molecular-weight (LMW) compounds self-assembled in different solvents via non-covalent interactions. In most cases, this feature enables reversible stimuli-responsive gel-to-sol transitions [31]. Usually, the entanglement of 1D nanofibers of gelator molecules generates a 3D network with the solvent molecules trapped in the interstices by means of surface tension and capillary forces. This provides the typical solid-like appearance and viscoelastic features to physical gels [32].

**Figure 1:**
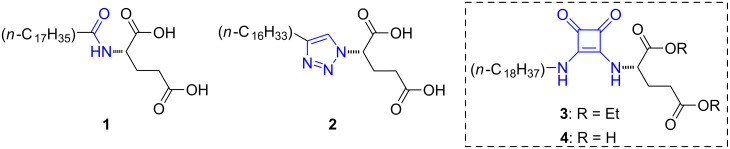
Chemical structures of isosteric gelators **1** and **2** previously studied [33], and squaramide-based analogues **3** and **4** investigated in this work.

Recently, we have demonstrated the potential of isosteric substitution for tuning the properties of supramolecular gels [33]. Specifically, we exchanged the amide group of *N*-stearoyl-L-glutamic acid (**1**, Figure 1), a known LMW gelator [34], by its non-classical isostere [35–36] 1,4-disubstituted 1,2,3-triazole **2** (Figure 1). This approach enabled us to fine-tuning the gelation capacity and the properties of the gels obtained with these compounds. In general, compound **2** formed gels in more solvents, at lower concentration and faster than compound **1**.

In this work, and based on our previous experience with organogels based on squaramides [21], we decided to prepare and study an analogue of *N*-stearoyl-L-glutamic acid bearing the squaramide moiety instead of the amide group (**4**, Figure 1). Interestingly, the gelation properties of the diester precursor **3** (Figure 1) was found to be superior than **4**, allowing to obtain a variety of gels at lower concentration than those obtained with **2**, as well as to form gels in some solvents where both gelators **1** and **2** failed.

## Results and Discussion

### Synthesis of squaramide-based gelators

**Scheme 1:**
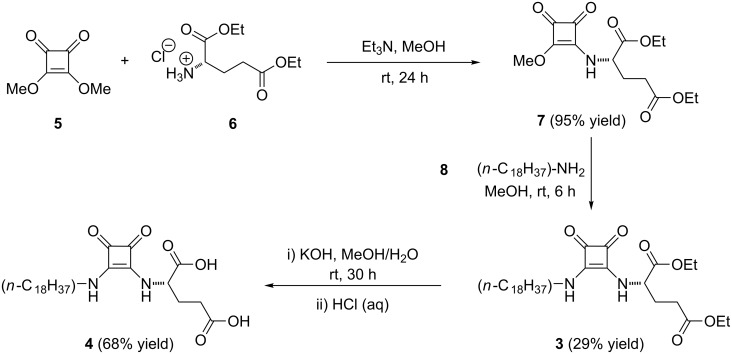
Synthesis of squaramide-based gelators **3** and **4**.

Squaramides are typically synthesized under mild conditions via aliphatic amine condensation of dialkoxysquarate derivatives [3]. In general, the use of an excess of aliphatic amines affords the corresponding symmetrical squaramides. However, we employed a two-step synthetic protocol in order to obtain the target unsymmetrical squaramide **3** (Scheme 1). The first step involved the reaction between L-glutamic acid diethyl ester hydrochloride (**6**) and dimethyl squarate (**5**) in the presence of Et_3_N in MeOH at room temperature (rt). The use of Et_3_N (1 equiv) and a low excess of **5** (1.1 equiv) gave the intermediate squarate monoamine **7** in 95% isolated yield. In the second step, compound **7** was subjected to a second reaction with *n*-octadecylamine (**8**) in MeOH at rt, affording the desired unsymmetrical squaramide **3** in a moderate yield of 29% after isolation. Then, the diester groups in **3** were hydrolyzed using an excess of KOH in a MeOH/H_2_O mixture, which allows us to obtain the desired diacid-containing squaramide **4** in 68% isolated yield upon acidification (pH 2).

### Gelation properties

The gelation capacities of squaramides **3** and **4** were screened for 22 different solvents of different nature (apolar, polar aprotic, polar protic) using the standard heating–cooling cycle (Table 1, Table 2 and Supporting Information File 1, Figure S1). Diester **3** was found to be soluble in methylene chloride, chloroform, tetrahydrofuran, xylene, toluene, benzene, and chlorobenzene, whereas it was insoluble in water even after heating. In contrast, gel materials that did not flow upon inversion of the vial upside-down were obtained in 12 solvents with critical gelation concentration (CGC) values ranging from 16 ± 1 to 180 ± 20 g/L (Table 1). CGC was defined as the minimum concentration of gelator where gelation was observed. Most gels were formed within 30 min and all of them showed a white opaque appearance, suggesting the formation of supramolecular aggregates larger than the range of visible light (380–780 nm), which was later supported by electron microscopy (see below). Moreover, the gels displayed full thermoreversibility and remained stable for at least two months when stored in sealed vials. Only the gel made in DMF showed a gradual gel-to-crystal transition [37–38], which is not surprising due to the delicate equilibrium between metastable gel and thermodynamically stable crystalline phases [39–40].

**Table 1:** Gelation ability, CGC, gelation time, *T*_gel_ and appearance of gels made of squaramide diester derivative **3**.^a^

solvent	CGC (g/L)	gelation time (min)	*T*_gel_ (°C)	appearance^b^

acetone	45 ± 5	19 ± 1	26 ± 2	opaque gel
acetonitrile	62 ± 5	0.4 ± 0.1	52 ± 2	opaque gel
benzonitrile	180 ± 20	4.3 ± 0.1	34 ± 2	opaque gel
butan-1-ol	56 ± 5	25 ± 5	34 ± 2	opaque gel
dimethyl sulfoxide	25 ± 1	28.5 ± 0.2	30 ± 2	opaque gel
ethanol	27 ± 2	7.9 ± 1.1	33 ± 2	opaque gel
ethoxyethane	47 ± 3	12.1 ± 0.9	42 ± 2	opaque gel
ethyl acetate	36 ± 4	2.8 ± 0.9	36 ± 2	opaque gel
hexan-1-ol	33 ± 1	77 ± 10	31 ± 2	opaque gel
methanol	47 ± 3	1.6 ± 0.1	41 ± 2	opaque gel
nitromethane	16 ± 1	3.5 ± 0.4	41 ± 2	opaque gel
propan-2-ol	33 ± 1	15.8 ± 1.4	29 ± 2	opaque gel

^a^Gels were obtained upon a heating–cooling cycle. Error values reported as standard deviation were estimated from at least two randomized experiments. ^b^Gels were white in color except the gel in nitromethane that was yellowish.

Squaramide **3** formed stable gels in the same number of solvents than the analogue triazole-based gelator **2**, which was previously found to be superior to the amide **1** [33]. Similarly to **3**, CGC values were established for **2** in a range of 10–200 g L^−1^. Gelator **3** formed stable gels in some solvents such as ethyl acetate and acetone, which were not gelled by **2**. In addition, **3** also formed gels in 1-hexanol and benzonitrile, solvents in which **2** only formed partial gels at *c* > 200 g/L. On the other hand, solvents such as methylene chloride, chloroform, xylene, benzene and toluene were gelled by **2** but not by **3**. Thus, compound **3** can be an alternative to compound **2**, and vice versa, depending on the solvent to be gelled (see below). Similarly to other amphiphilic gelators [21,29,31], the formation of a self-assembled network in organic solvents is likely driven by the formation of hydrogen bonds between different gelator molecules (polar head) as well as hydrophobic interactions between the long aliphatic chains.

Considering our previous results obtained with diacids **1** and **2** [33], we initially expected a good gelation ability of the diacid squaramide **4**. To our surprise, **4** showed a very limited gelation capacity (Table 2) in comparison with its diester precursor **3** (Table 1). Four gels made of **4** were formed in chloroform, methanol, propan-2-ol and toluene. However, it should be noted that two of these solvents (chloroform and toluene) were not gelled by **3**. Moreover, it is worth mentioning that these results do not discard the possibility of obtaining additional gels using higher concentrations of **4** and/or resting time than those established for this study (see Experimental section).

**Table 2:** Gelation ability, CGC, gelation time, *T*_gel_ and appearance of gels made of squaramide diacid derivative **4**.^a^

solvent	CGC (g/L)	gelation time (min)	*T*_gel_ (ºC)	appearance^b^

chloroform	38 ± 2	0.6 ± 0.1	42 ± 1	opaque gel
methanol	117 ± 17	2.4 ± 0.4	29 ± 1	opaque gel
propan-2-ol	200 ± 5	4.6 ± 0.1	34 ± 6	opaque gel
toluene	50 ± 1	0.8 ± 0.1	28 ± 2	translucent gel

^a^Gels were obtained upon a heating–cooling cycle. Error values reported as standard deviation were estimated from at least two randomized experiments. ^b^The gel in chloroform was white in color. The rest of the gels were yellowish.

Although in this work all gels were prepared via heating–cooling, we observed that the application of ultrasound [41] facilitated the formation of some gels by decreasing significantly the gelation time, especially after the gels were thermally destroyed for the first time (data not shown). This is in good agreement with our previous observations made with a different squaramide, where we hypothesized that ultrasound could help to preserve only the thermodynamically more stable aggregates through a self-sorting mechanism, thus providing a more robust starting platform for rebuilding the gel network [21].

At this point, we decided to perform some additional studies focusing on the gels made of diester **3** due to its apparent higher versatility with regard to gelation scope. For instance, the comparison of Fourier transform infrared (FTIR) spectra of a gel made of **3** and its solution did not show frequency shifts for characteristic bands such as C=O stretching (≈1735 cm^−1^), and N–H stretching ≈2867–3000 cm^−1^ (Supporting Information File 1, Figure S2). This suggests that the gelator may also be aggregated in solution, at least to some extend, via similar hydrogen-bonding interactions than in the gel state. In contrast, the spectrum of the xerogel, prepared by freeze-drying the corresponding organogel, revealed a red shift (lower frequency) of the above-mentioned stretching bands compared to solid **3** (C=O Δν ≈5 cm^−1^; N–H Δν ≈70 cm^−1^), which is an indication of increased hydrogen-bonding.

### Characterization of organogels

**Figure 2:**
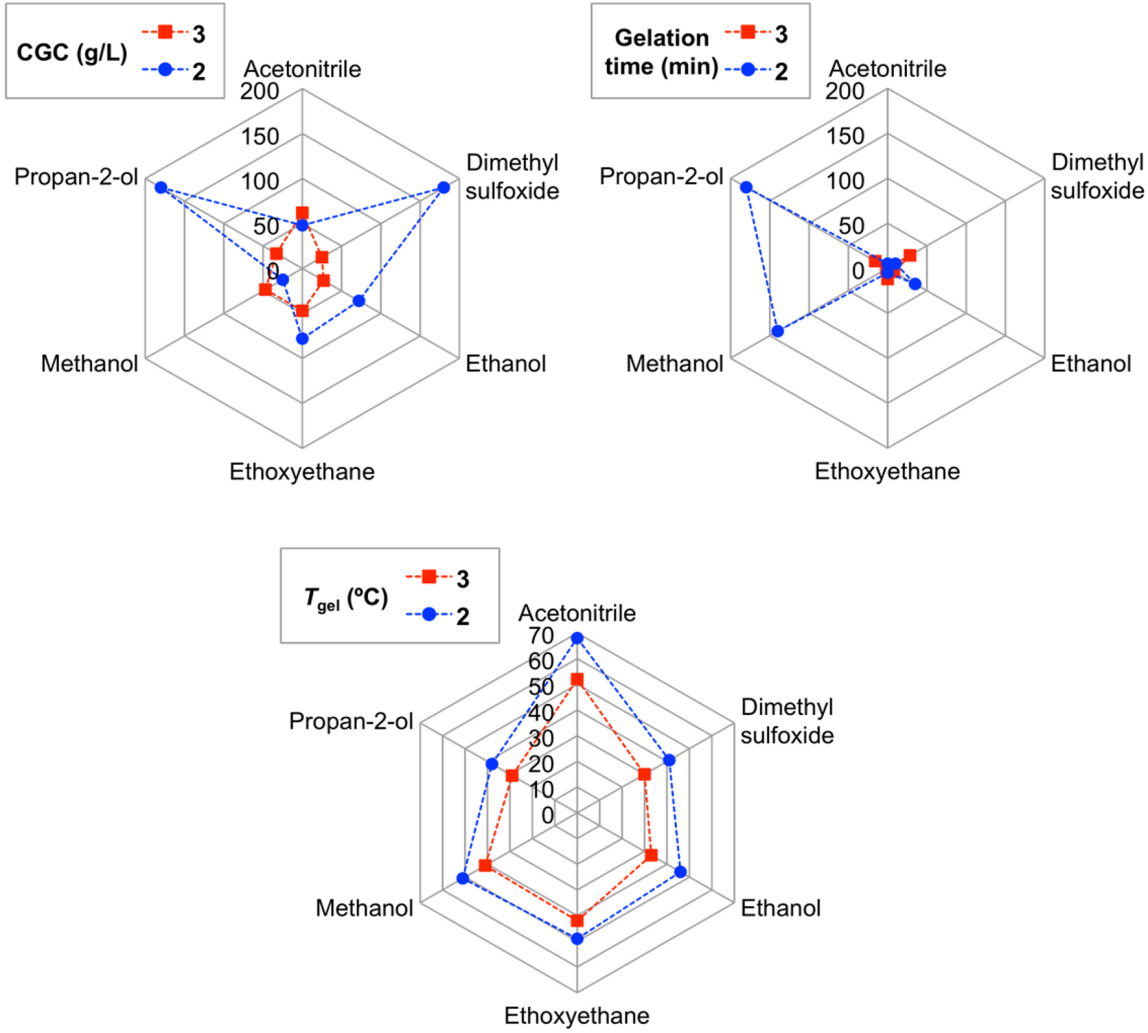
Comparison of CGC, gelation time and *T*_gel_ values corresponding to six gels made using **3** and **2** [33] as gelators.

In general, all gels displayed relatively low gel-to-sol transition temperatures (*T*_gel_) ranging from 26 to 52 °C (±2, Table 1). A comparative study made with a population of six gels showed that the *T*_gel_ of the gels made of **3** were in general ca. 10 °C lower than those obtained using gelator **2** (Figure 2). On the positive side, lower CGC values and gelation times were generally achieved when using squaramide **3** as gelator.

**Figure 3:**
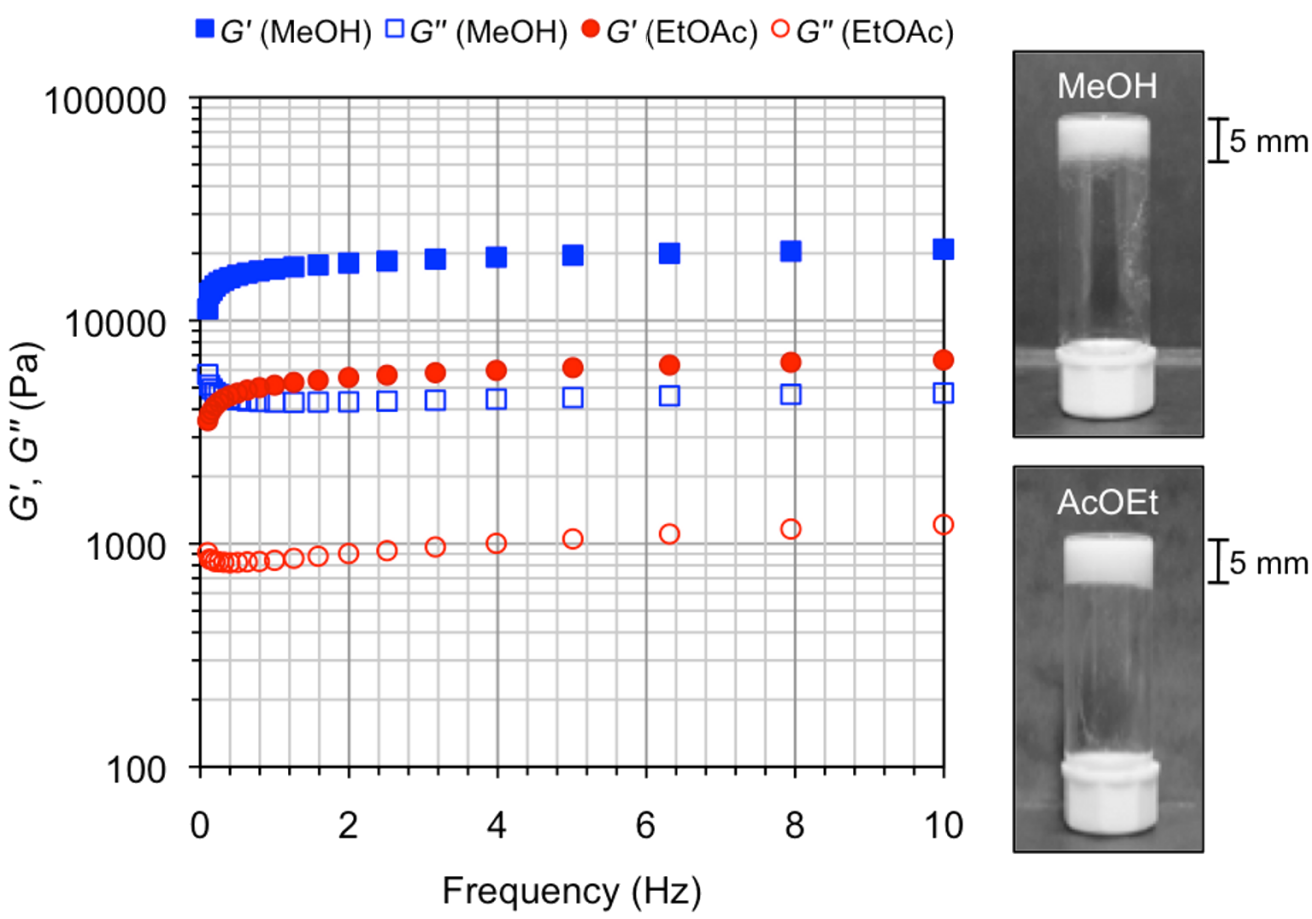
DFS measurements for model gels made of **3** in methanol (*c* = 47 g/L) and ethyl acetate (*c* = 36 g/L). Photographs on the right correspond to upside-down vials having the selected gels.

In order to confirm the viscoelastic nature of gels, we performed oscillatory rheological measurements on two representative gels made of **3** in methanol and ethyl acetate. The linear regime was established by dynamic frequency sweep (DFS) and dynamic strain sweep (DSS) experiments (see Experimental section). The results showed that the storage modulus *G'* of both gels was one order of magnitude higher than the respective loss modulus *G''*, maintaining a relatively low frequency dependency (i.e., gel in methanol: *G'* ≈ 17 ± 0.1 kPa, *G''* ≈ 4.6 ± 0.1 kPa, *G* ≈ ν^0.11–0.03^; gel in ethyl acetate: *G'* ≈ 5.1 ± 0.1 kPa, *G''* ≈ 0.92 ± 0.1 kPa, *G* ≈ ν^0.13–0.07^, Figure 3). The damping coefficient or loss factor (tan δ = *G’’*/*G’*) of the gel in methanol was about 1.5 times higher than that of the gel in ethyl acetate, indicating higher energy dissipation potential for the former. Moreover, both gels were brittle in nature as confirmed by destruction at low frequency and ≈4 ± 0.3% of strain (Supporting Information File 1, Figure S3).

**Figure 4:**
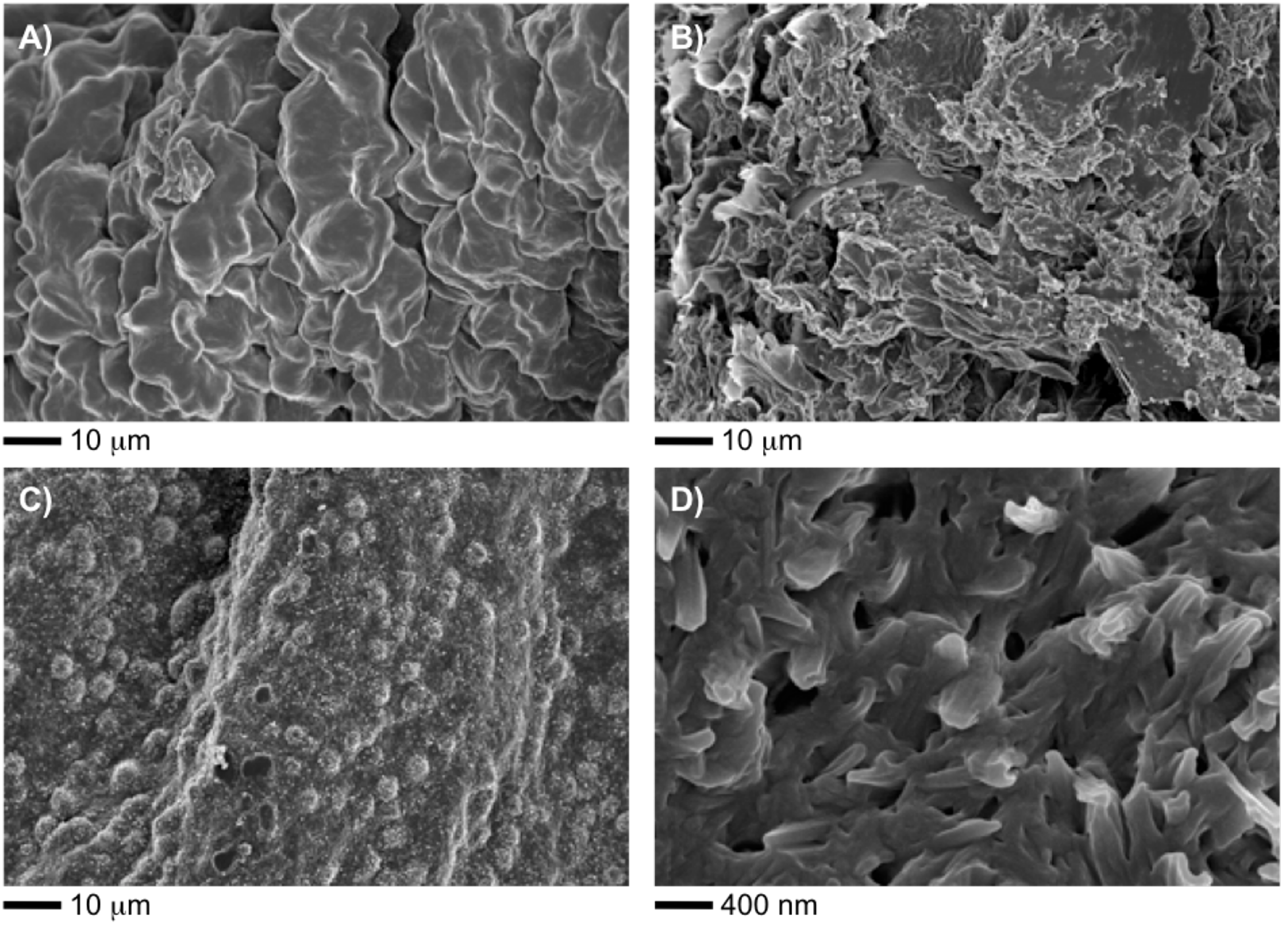
Representative FESEM images of selected xerogels prepared by freeze-drying the corresponding organogels made of **3** in (A) ethyl acetate (*c* = 36 g/L), (B) methanol (*c* = 47 g/L), (C and D) butan-1-ol (*c* = 56 g/L).

Morphological studies of some selected organogels were conducted by field emission scanning electron microscopy (FESEM) of the corresponding xerogels obtained by the freeze-drying method (Figure 4). The remarkable influence of the solvents on the morphologies was evident among different samples. For instance, the specimens prepared in ethyl acetate showed an entangled brain coral-like structure (Figure 4A), whereas the xerogel made in methanol displayed a less regular wrinkled lamellar-like structure (Figure 4B). Interestingly, the use of 1-butanol instead of methanol afforded a xerogel characterized by a poritidae-like porous structure formed by numerous fibrillar and globular structures of ca. 2–4 μm in diameter (Figure 4C, D). Although the recorded images correspond to the bulk material, it should be stressed that the formation of artifacts during the drying process can not be completely ruled out [42]. Hence, the interpretation of these images should always be done cautiously. Further detailed investigations with the aid of additional techniques are still necessary in order to clarify the exact molecular mechanism associated with each morphology.

## Conclusion

In conclusion, unsymmetrical glutamic acid-based squaramides **3** and **4** can be synthesized for subsequent amine condensations starting from dimethyl squarate. These compounds were found to self-assemble in different organic solvents leading to the formation of stable supramolecular gels upon a classical heating–cooling cycle. Thus, these two compounds expand the short list of squaramide-based LMW gelators reported so far in the literature. As LMW gelator, squaramide diester **3** was found to be superior than the corresponding diacid **4** (i.e., **3** formed stable gels in 12 solvents, whereas **4** only gelled four solvents). CGC values ranged from 16 ± 1 to 180 ± 20 g/L for **3** and from 38 ± 2 to 200 ± 5 g/L for **4**. In terms of *T*_gel_ the values ranged from 26 ± 2 to 52 ± 2 °C for **3** and from 29 ± 1 to 42 ± 1 °C for **4**. Further rheological and electron microscopy studies of selected gels demonstrated their viscoelastic nature as well as the remarkable influence of the solvents on their flow properties and microstructures. Finally, a comparison between **3** and a previously studied analogue triazole-based gelator **2** showed that both gelators can gel 6 solvents in common. However, some other solvents are only gelled by either **3** (i.e., ethyl acetate, acetone) or **2** (i.e., methylene chloride, chloroform, xylene, benzene and toluene). Moreover, **3** also formed gels in 1-hexanol and benzonitrile, solvents in which **2** only formed partial gels at *c* > 200 g/L. Although the *T*_gel_ of the gels made of **3** were ca. 10 ºC lower than those obtained using gelator **2**, lower CGC values and gelation times were generally achieved when using squaramide **3** as gelator.

## Experimental

### Synthesis of compounds

#### General remarks

Unless otherwise specified, all reagents, starting materials and solvents (p.a. grade) were purchased from commercial suppliers and used as received without further purification.

#### Characterization methods

Thin-layer chromatography (TLC) analyses were performed using fluorescent-indicating plates (aluminum sheets coated with silica gel 60 F_254_, thickness 0.2 mm, Merck). Visualization was achieved by UV light (λ_max_ = 254 nm). Melting point calculations were made using a GallenKamp MPD 350 BM 2.5 instrument. Specific rotation calculations were made in chloroform or acetone employing a Jasco P-1020 polarimeter. ESI ionization method and mass analyzer type MicroTof-Q were used for HRMS measurements. ^1^H NMR spectra and ^13^C APT-NMR spectra were recorded at 300 MHz and 75 MHz, respectively, using a Bruker ARX 300 MHz spectrometer. CDCl_3_ and DMSO-*d*_6_ were used as deuterated solvents. Chemical shifts were reported in the δ scale relative to residual CHCl_3_ (7.26 ppm) and DMSO (2.50 ppm) for ^1^H NMR and to the central line of CDCl_3_ (77.16 ppm) and DMSO-*d*_6_ (39.52 ppm) for ^13^C-APT-NMR.

#### Synthetic procedures and characterization data

**(*S*)-Diethyl 2-((2-methoxy-3,4-dioxocyclobut-1-en-1-yl)amino)pentanedioate (7):** L-Glutamic acid diethyl ester hydrochloride (**6**, 3.6 g, 15 mmol) was dissolved in MeOH (30 mL) and Et_3_N (2.1 mL, 15 mmol) was added dropwise. The resulting solution was added dropwise to a mixture of 3,4-dimethoxy-3-cyclobutene-1,2-dione (**5**, 2.4 g, 16.5 mmol) in MeOH (30 mL) at room temperature. After 24 h, the solvent was removed under vacuum and the product was purified by column chromatography (SiO_2_, hexane/EtOAc 7:3 to hexane/EtOAc 1:1). Product **7** was obtained as a brown oil in 95% yield (4.5 g, 14.25 mmol); [α]_D_^22^ +11.6 (*c* 0.56, CHCl_3_); ^1^H NMR (300 MHz, DMSO-*d*_6_) δ 9.10 (d, *J* = 7.6 Hz, 0.5H, NH), 8.88 (d, *J* = 7.9 Hz, 0.5H, NH), 4.70–4.58 (m, 0.5H, NH-C*H*), 4.40–3.98 (m, 7.5H, O-CH_3_, NH-C*H* and O=C-O-CH_2_), 2.48–2.33 (m, 2H, NH-CH-CH_2_-C*H*_2_), 2.25–2.05 (m, 1H, NH-CH-C*H*_2_), 2.02–1.85 (m, 1H, NH-CH-C*H*_2_), 1.18 (t, *J* = 7.1 Hz, 6H, O=C-O-CH_2_-C*H*_3_); ^13^C APT-NMR (75 MHz, DMSO-*d*_6_) δ 189.1 and 188.7 (2 s, 1C, O=*C*-C=C-C=O or O=C-*C*=C-C=O), 183.1 and 182.8 (2 s, 1C, O=*C*-C=C-C=O or O=C-*C*=C-C=O), 177.9 and 177.8 (2 s, 1C, O=*C*-C=C-C=O or O=C-*C*=C-C=O), 172.8 and 172.3 (2 s, 1C, O=C-*C*=C-C=O), 171.9 (s, 1C, O=C-O), 170.6 and 170.3 (2 s, 1C, O=C-O), 61.3 (s, 1C, O=C-O-*C*H_2_), 60.3 and 60.1 (2 s, 1C, O-CH_3_), 60.0 (s, 1C, O=C-O-*C*H_2_), 55.8 and 55.2 (2 s, 1C, NH-CH), 29.7 (s, 1C, NH-CH-CH_2_-*C*H_2_), 26.8 and 26.5 (s, 1C, NH-CH-*C*H_2_), 14.0 (s, 1C, O=C-O-CH_2_-*C*H_3_), 14.0 (s, 1C, O=C-O-CH_2_-*C*H_3_); FTIR (oil, cm^−1^) ν: 3269, 2983, 1806, 1736, 1653, 1618, 1610, 1500, 1464, 1378, 1345, 1299, 1263, 1201, 1102, 1024; HRMS (ESI^+^) *m*/*z*: [M + Na]^+^ calcd for C_14_H_19_NNaO_7_, 336.1054; found, 336.1094.

**(*S*)-Diethyl 2-((2-(octadecylamino)-3,4-dioxocyclobut-1-en-1-yl)amino)pentanedioate (3):** A solution of *n*-octadecylamine (**8**, 3.9 g, 14.5 mmol) in MeOH (60 mL) was added to a mixture of squarate monoamine **7** (4.5 g, 14.5 mmol) in MeOH (160 mL) at room temperature. After 6 h, the solvent was removed under vacuum and the product was purified by column chromatography (SiO_2_, hexane/EtOAc 8:2 to hexane/EtOAc 6:4). Then, the solvent from the column was evaporated and the yellowish solid obtained was washed with 15 mL EtOAc (× 3). After this, squaramide **3** was obtained as a white solid in 29% yield (2.3 g, 4.2 mmol); mp 79–81 °C; [α]_D_^27^ +11.0 (*c* 0.50, CHCl_3_); ^1^H NMR (300 MHz, CDCl_3_) δ 7.86 (br s, 1H, NH), 4.91 (br s, 0.5H, NH-C*H*), 4.21 (br s, 1H, NH-C*H*_2_), 3.87–3.46 (m, 4.5H, NH-C*H* and O=C-O-CH_2_), 3.08 (br s, 1H, NH-C*H*_2_), 2.68–1.95 (m, 4H, NH-CH-C*H*_2_ and NH-CH-CH_2_-C*H*_2_), 1.67 (br s, 2H, NH-CH_2_-C*H*_2_), 1.51–0.96 (m, 36H, -CH_2_), 0.92–0.81 (m, 3H, -CH_3_); ^13^C APT-NMR (75 MHz, CDCl_3_) δ 181.7 (s, 1C, O=*C*-C=C-C=O or O=C-*C*=C-C=O), 173.3 (s, 1C, O=*C*-C=C-C=O or O=C-*C*=C-C=O), 171.7 (s, 1C, O=C-O), 171.2 (s, 1C, O=C-O), 168.7 (s, 1C, O=*C*-C=C-C=O or O=C-*C*=C-C=O), 166.2 (s, 1C, O=*C*-C=C-C=O or O=C-*C*=C-C=O), 62.2 (s, 1C, O=C-O-*C*H_2_), 60.6 (s, 1C, O=C-O-*C*H_2_), 56.0 and 52.1 (2 s, 1C, NH-CH), 45.2 (s, 1C, NH-CH_2_), 32.1 (s, 1C, -CH_2_), 31.3 (s, 1C, -CH_2_), 30.1 (s, 1C, -CH_2_), 29.9 (s, 10C, -CH_2_), 29.8 (s, 1C, -CH_2_), 29.5 (s, 1C, -CH_2_), 29.5 (s, 1C, -CH_2_), 26.7 (s, 1C, -CH_2_), 22.8 (s, 1C, -CH_2_), 14.4 (s, 1C, O=C-O-CH_2_-*C*H_3_), 14.3 (s, 1C, O=C-O-CH_2_-*C*H_3_), 14.3 (s, 1C, -CH_3_); FTIR (solid, cm^−1^) ν: 2916, 2849, 1799, 1728, 1613, 1557, 1464, 1375, 1203, 1021, 720; HRMS (ESI^+^) *m/z*: [M + Na]^+^ calcd for C_31_H_54_N_2_O_6_Na, 573.3874; found, 573.3840.

**(*S*)-2-((2-(Octadecylamino)-3,4-dioxocyclobut-1-en-1-yl)amino)pentanedioic acid (4):** Squaramide **3** (1.2 g, 2.15 mmol) was dissolved in a 1:1 v/v MeOH/H_2_O mixture (40 mL) containing KOH (0.36 g, 6.45 mmol) at room temperature. After 30 h, the solution was acidified with HCl (1 M) until pH 2 was reached. Then, the solid was filtrated and washed with 5 mL of water (× 3) and 5 mL of CHCl_3_. Finally, the solid was dried in the oven at 80 °C for 1 h, affording the squaramide **4** as a brown solid in 68% yield (0.72 g, 1.46 mmol); mp 115–120 °C; [α]_D_^20^ +10.3 (*c* 0.27, EtOH); ^1^H NMR (300 MHz, DMSO-*d*_6_) δ 12.65 (br s, 2H, CO_2_H), 7.64 (d, *J* = 7.2 Hz, 1H, C=C-N*H*-CH), 7.51 (br s, 1H, C=C-N*H*-CH_2_), 4.78–4.47 (m, 1H, NH-C*H*), 3.68–3.38 (m, 2H, NH-C*H*_2_), 2.38–2.19 (m, 2H, NH-CH-CH_2_-C*H*_2_), 2.17–2.02 (m, 1H, NH-CH-C*H*_2_), 1.99–1.83 (m, 1H, NH-CH-C*H*_2_), 1.60–0.94 (m, 32H, -CH_2_), 0.92–0.75 (m, 3H, -CH_3_); ^13^C APT-NMR (75 MHz, DMSO-*d*_6_) δ 183.0 (s, 1C, O=*C*-C=C-C=O or O=C-*C*=C-C=O), 182.0 (s, 1C, O=*C*-C=C-C=O or O=C-*C*=C-C=O), 173.4 (s, 1C, O=C-OH), 172.7 (s, 1C, O=C-OH), 168.0 (s, 1C, O=*C*-C=C-C=O or O=C-*C*=C-C=O), 167.0 (s, 1C, O=*C*-C=C-C=O or O=C-*C*=C-C=O), 55.0 (s, 1C, NH-CH), 43.3 (s, 1C, NH-CH_2_), 31.3 (s, 1C, -CH_2_), 30.6 (s, 1C, -CH_2_), 29.7 (s, 1C, -CH_2_), 29.0 (s, 11C, -CH_2_), 28.7 (s, 1C, -CH_2_), 28.6 (s, 1C, -CH_2_), 25.8 (s, 1C, -CH_2_), 22.1 (s, 1C, -CH_2_), 13.9 (s, 1C, -CH_3_); FTIR (solid, cm^−1^) ν: 2923, 2852, 1743, 1653, 1579, 1466, 1377, 1147, 839, 720. HRMS (ESI^+^) *m*/*z*: [M + Na]^+^ calcd for C_27_H_46_N_2_O_6_Na, 517.3248; found, 517.3217.

#### Preparation and characterization of gel materials

Gels were prepared in screw-capped glass vials (4.5 cm length × 1 cm diameter) having a specific amount of the desired gelator and solvent (p.a. grade). The mixture was gently heated with a heat gun until the solid material was completely dissolved (i.e., a transparent solution without visible suspended particles was obtained). The resulting isotropic solution was allowed to cool down to rt affording the corresponding gels. No control over temperature rate during the heating–cooling process was applied. Double-distilled water was purified additionally using a Millipore water-purifying system (Merck) prior usage. Xylene as mixture of isomers was used after double-distillation.

CGC values were estimated by continuously adding aliquots of solvent (0.05−0.1 mL) into vials containing 20 mg of the gelator and performing a typical heating–cooling protocol for gel formation until no gelation was observed. The starting point for CGC determinations was 200 g/L. The waiting time used to define the state of the material was 24 h.

*T*_gel_ values were determined using a calibrated thermoblock at a heating rate of ca*.* 5 °C/min [20]. The temperature at which the gel started to break was defined as *T*_gel_. Each measurement was made at least by duplicate and the average value reported.

FTIR spectra were recorded at rt using an Excalibur FTS 3000 FTIR spectrometer (Biorad) equipped with an attenuated total reflection (ATR) accessory (Golden Gate, Diamond).

Oscillatory rheological measurements were performed with an AR 2000 Advanced rheometer (TA Instruments) equipped with a Julabo C cooling system. A 500 μm gap setting and a torque setting of 5 × 10^−4^ N/m at 25 °C were used for the measurements in a plain-plate geometry (40 mm, stainless steel). 2 mL of the desired gel was taken carefully with a spatula and spread over the entire area of the plate without losing liquid. The following experiments were carried out for each sample: a) Dynamic strain sweep (DSS): variation of *G'* and *G''* with strain (from 0.01 to 100%); b) dynamic frequency sweep (DFS): variation of *G'* and *G''* with frequency (from 0.1 to 10 Hz at 0.1% strain).

FESEM of xerogels was carried out with a Carl Zeiss Merlin, Field Emission Scanning Electron Microscope (accelerating voltage = 10 kV). Xerogels, prepared by freeze-drying the corresponding gels, were placed on top of a tin plate and shielded with Pt (40 mA, 30–60 s; film thickness = 5–10 nm). Images were obtained by Servicio General de Apoyo a la Investigación-SAI (Universidad de Zaragoza).

## Supporting Information

File 1 NMR spectra, FTIR spectra, DSS plots, and additional photographs of gels in different solvents.
