# Supplementary material for: Synthesis and supramolecular self-assembly of glutamic acid-based squaramides
Source: Beilstein J Org Chem. 2018 Aug 6;14:2065–73. doi: 10.3762/bjoc.14.180 (PMC6122139; doi:10.3762/bjoc.14.180)
Supplement: File 1 — NMR spectra, FTIR spectra, DSS plots, and additional photographs of gels in different solvents. [file Beilstein_J_Org_Chem-14-2065-s001.pdf]

Supporting Information

for

Synthesis and supramolecular self-assembly of glutamic acid-based squaramides

Juan V. Alegre-Requena,[1] Marleen Häring,[2] Isaac G. Sonsona,[1] Alex Abramov,[2] Eugenia Marqués-López,[1] Raquel P. Herrera*[1] and David Díaz Díaz*[2,3]

Address: [1]Laboratorio de Organocatálisis Asimétrica. Departamento de Química Orgánica Instituto de Síntesis Química y Catálisis Homogénea (ISQCH) CSIC-Universidad de Zaragoza C/ Pedro Cerbuna 12, 50009 Zaragoza, Spain. [2]Institut für Organische Chemie, Universität Regensburg, Universitätsstr. 31, 93053 Regensburg, Germany and [3]Institute of Advanced Chemistry of Catalonia-Spanish National Research Council (IQAC-CSIC), Jordi Girona 18-26, 08034 Barcelona, Spain

Email: Raquel P. Herrera* - raquelph@unizar.es; David Díaz Díaz* - David.Diaz@chemie.uni-regensburg.de

*Corresponding author

NMR spectra, FTIR spectra, DSS plots, and additional photographs of gels in different solvents

List of contents

		page
1.	^{1}H NMR and ^{13}C NMR-APT spectra of new compounds …………………	S3
2.	Photographs of gel materials …………………………………………….…...	S6
3.	FTIR spectra ……………………………………………………………….	S7
4.	DSS rheological measurements …………………………………………….	S8

1. NMR spectra of new compounds

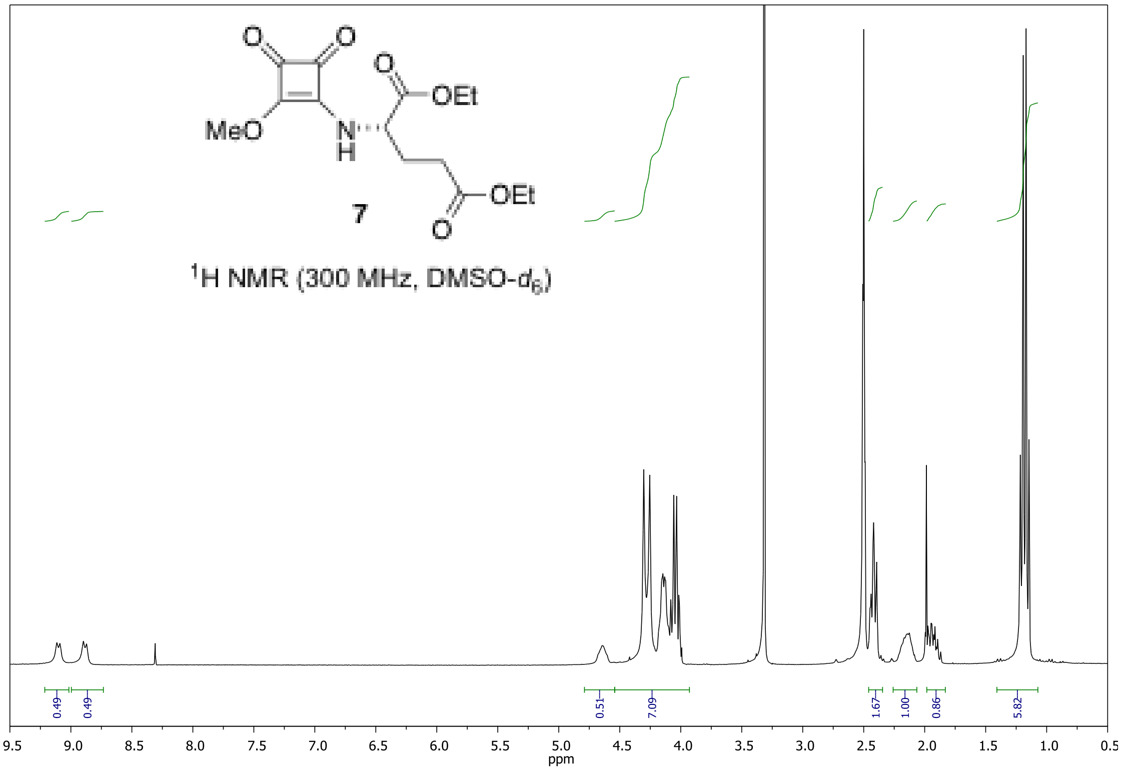

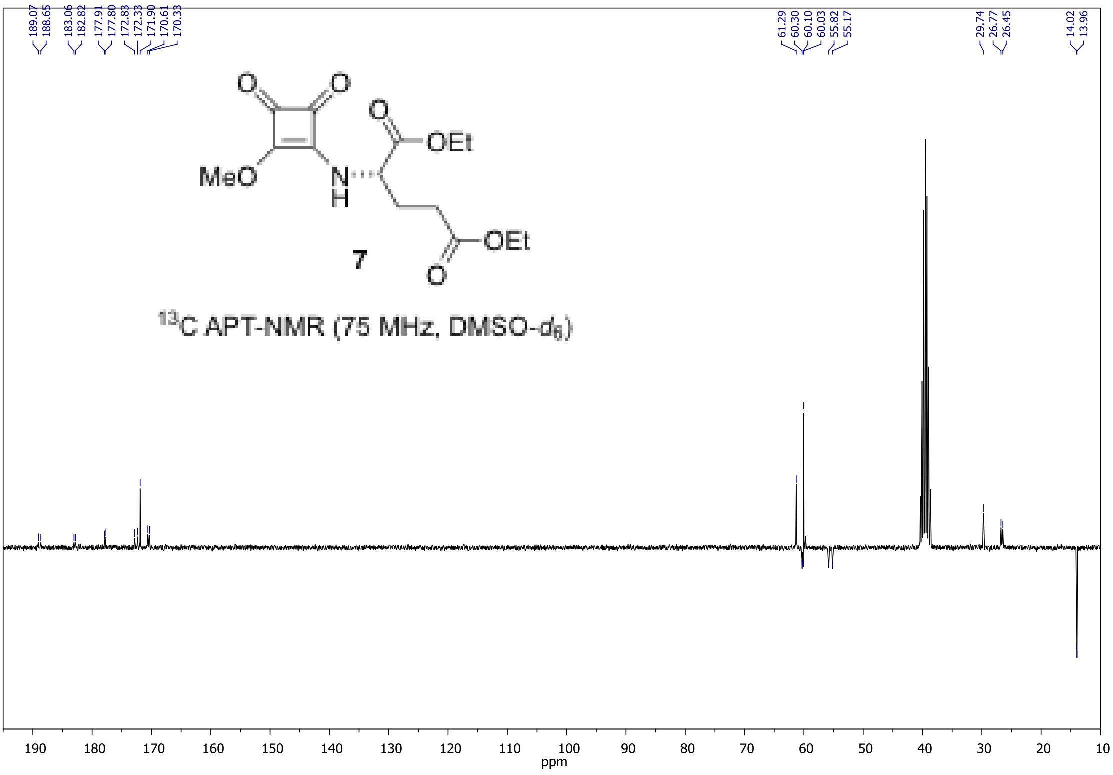

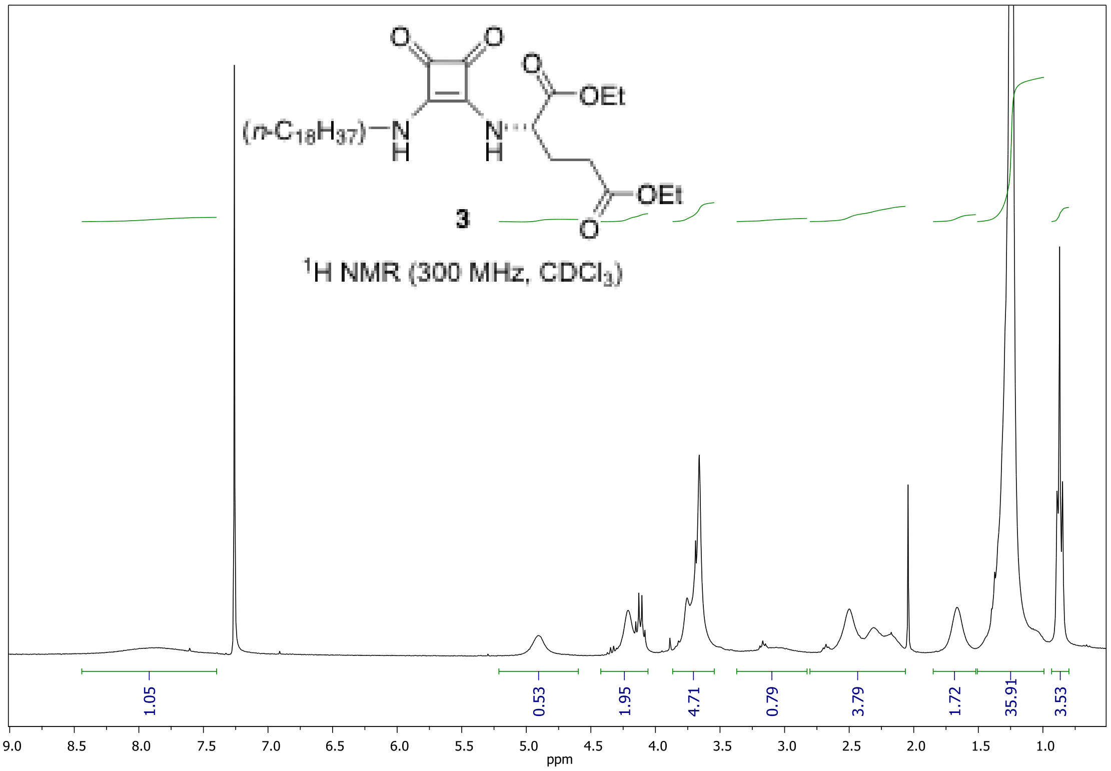

(n-C18H37)–N H
N H
OEt
OEt
3
1H NMR (300 MHz, CDCl3)
1.05
0.53
1.95
4.71
0.79
3.79
1.72
35.91
3.53
ppm

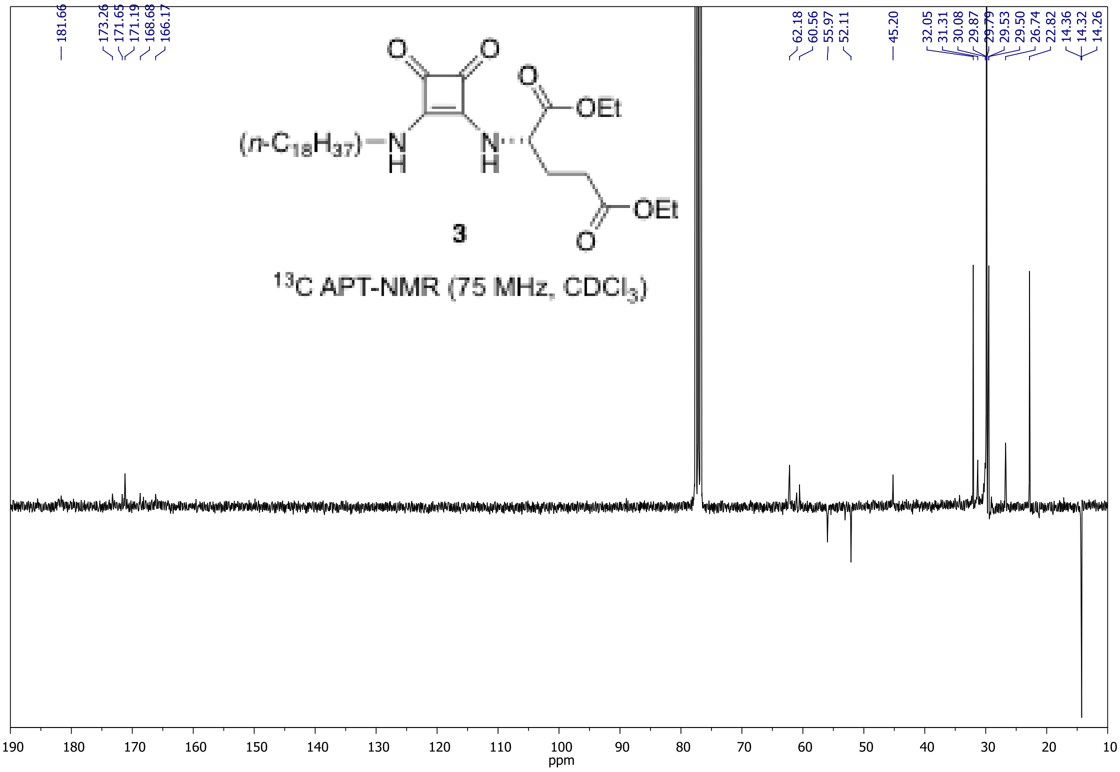

181.66
173.26
171.65
171.19
168.68
166.17
62.18
60.56
55.97
52.11
45.20
32.05
31.31
30.08
29.87
29.79
29.53
29.50
26.74
22.82
14.36
14.32
14.26
(n-C18H37)–N H
N H
OEt
OEt
3
13C APT-NMR (75 MHz, CDCl3)
ppm

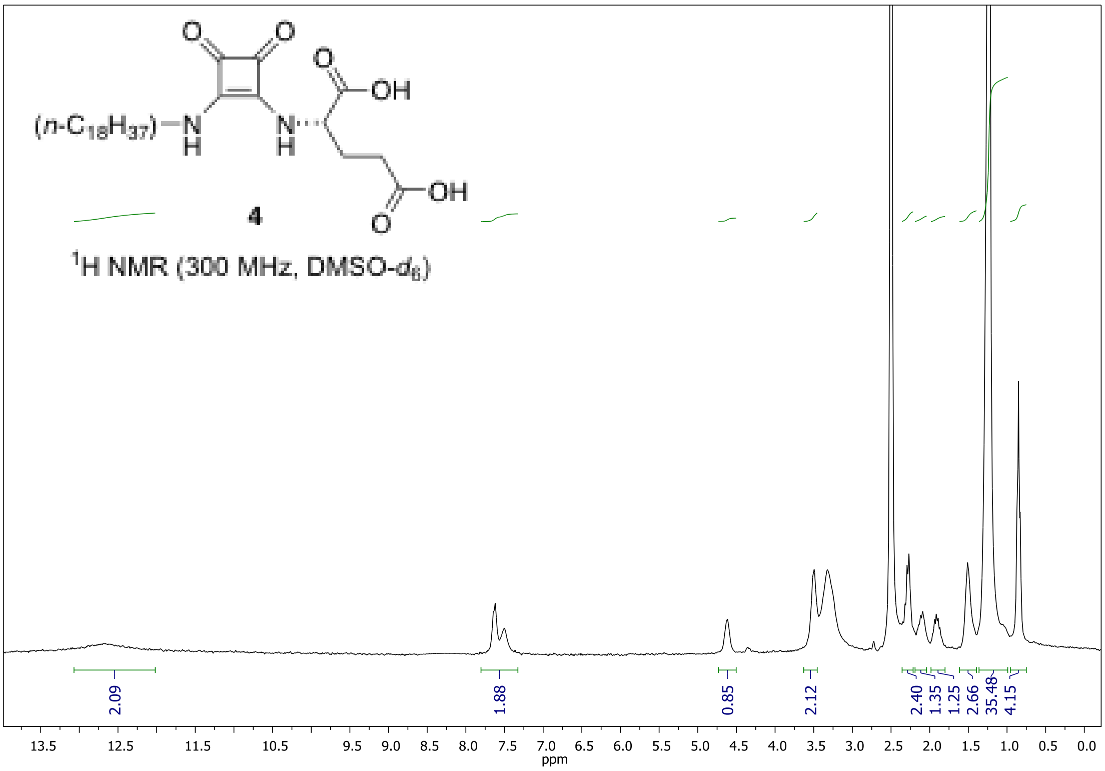

(n-C18H37)—N
H
N
H
OH
OH
4
1H NMR (300 MHz, DMSO-d6)
2.09
1.88
0.85
2.12
2.40
1.35
1.25
2.66
35.48
4.15
ppm

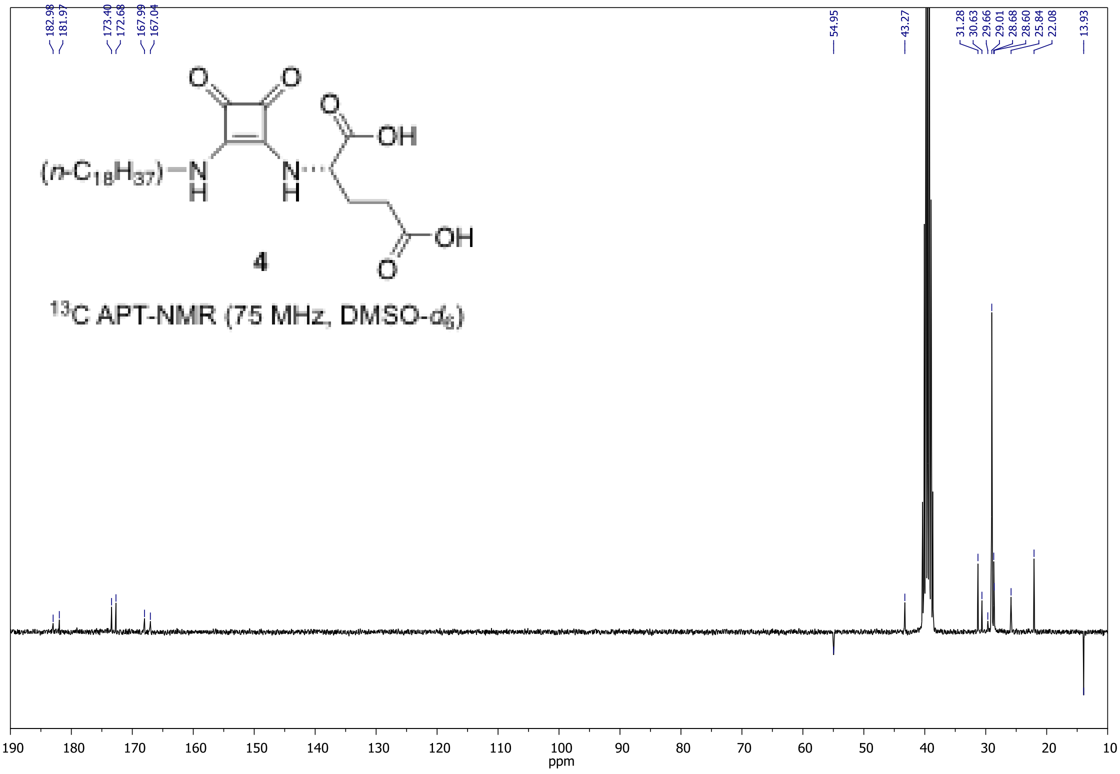

182.98
181.97
173.40
172.68
167.99
167.04
54.95
43.27
31.28
30.63
29.66
29.01
28.68
28.60
25.84
22.08
13.93
(n-C18H37)—N
H
N
H
OH
OH
4
13C APT-NMR (75 MHz, DMSO-d6)
ppm

2. Photographs of gel materials

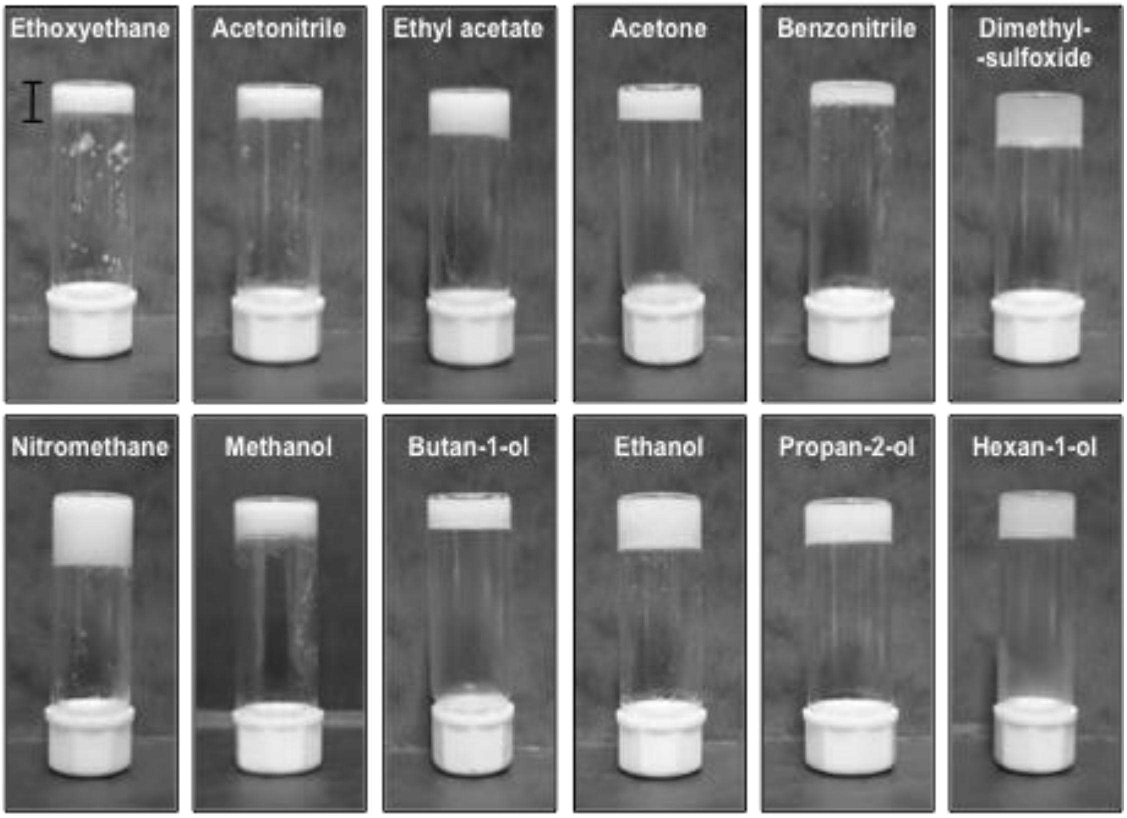

Figure S1: Selection of photographs showing upside-down vials containing gels made of **3** at their corresponding CGC (see Table 1) in different solvents. The scale bar included in the first photograph corresponds to 5 mm and it is the same for all photographs.

3. FTIR spectra

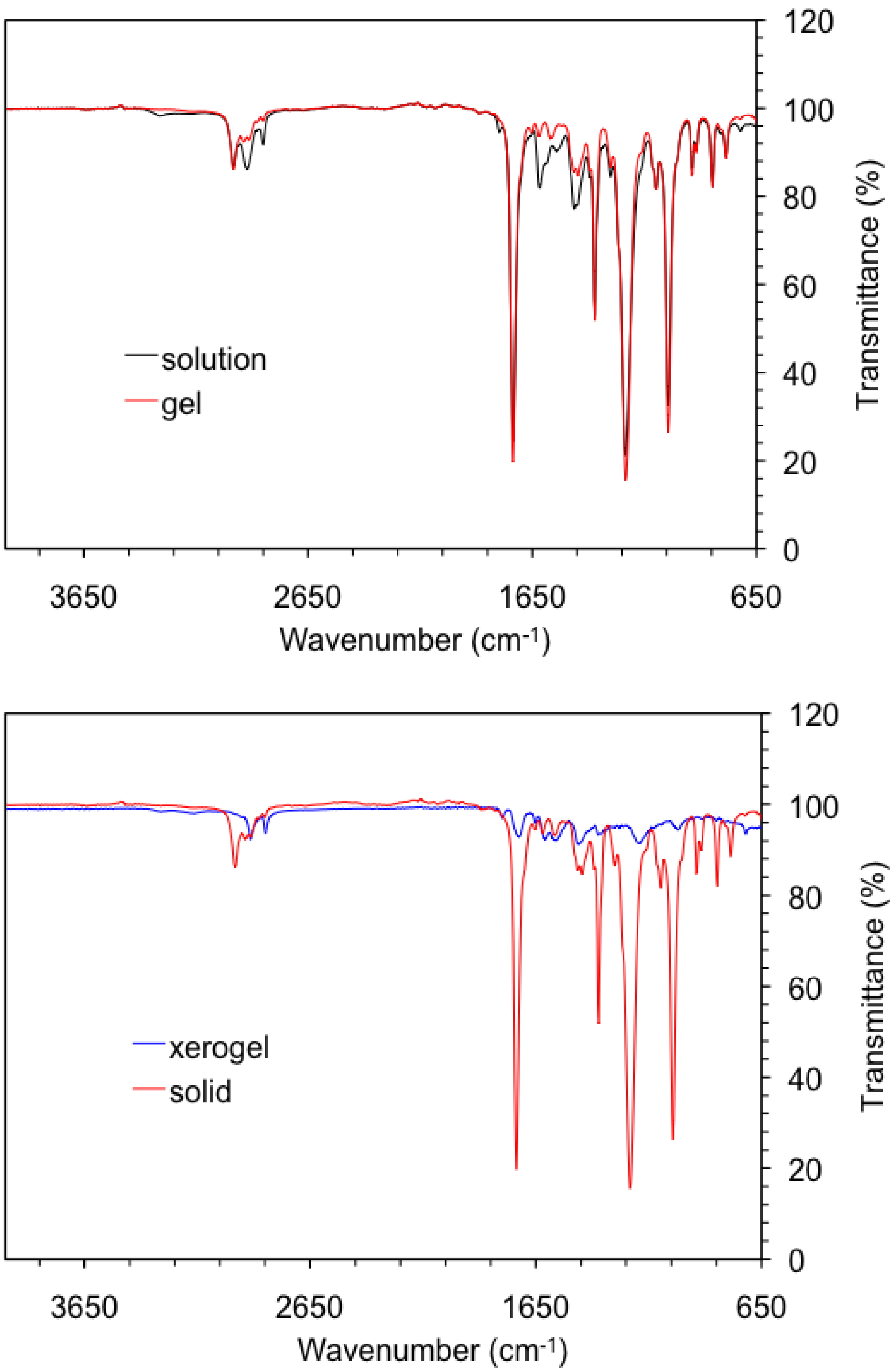

Figure S2: *Top*: FTIR spectra of the gel made of **3** in ethyl acetate (*c* = 36 g/L) and corresponding solution upon heating. *Bottom*: FTIR spectra of solid **3** (as synthesized) and xerogel obtained by freeze-drying the corresponding organogel made in ethyl acetate (*c* = 36 g/L).

4. DSS rheological measurements

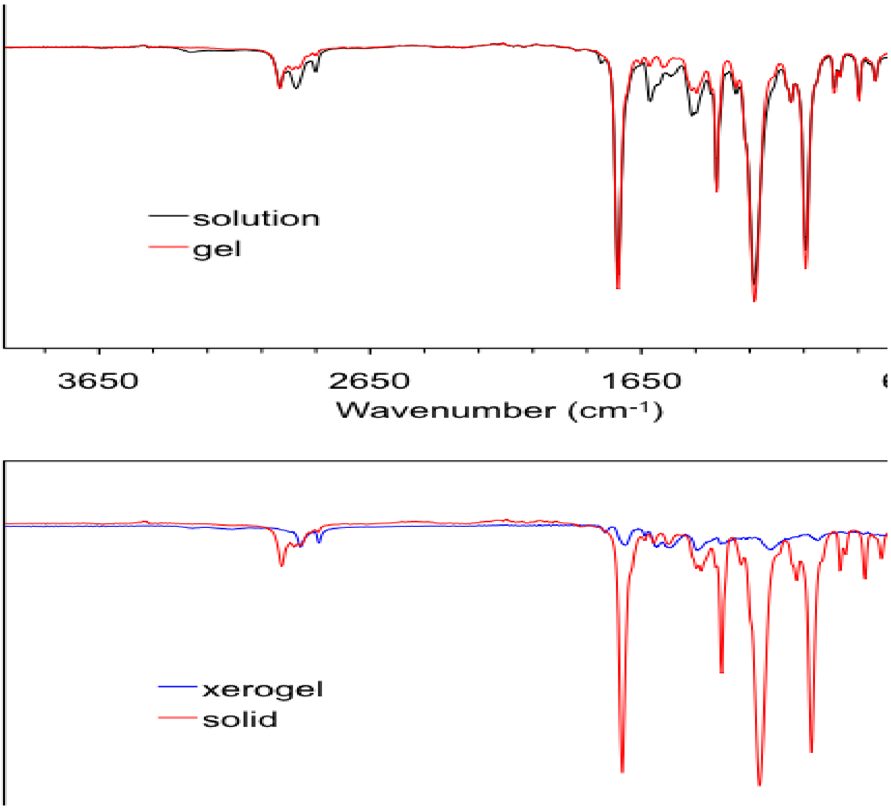

Figure S3: DSS measurements for model gels made of **3** in methanol (*c* = 47 g/L) and and ethyl acetate (*c* = 36 g/L). The tan δ values for these gels were 0.2716 and 0.1790, respectively. Color, CGC, T_{gel} and gelation kinetics were similar from batch to batch experiments (error values are given in the main paper).
